# Supplementary material for: Resolving the full spectrum of human genome variation using Linked-Reads
Source: Genome Res. 2019 Apr;29(4):635–45. doi: 10.1101/gr.234443.118 (PMC6442396; doi:10.1101/gr.234443.118)
Supplement: Supplemental Material [file supp_gr.234443.118_Supplemental_longranger-2.2.zip › longranger-2.2.tar.bz2!/lr2.2-public/lib/stan/cmdstan/stan/doc/api/html/classstan_1_1io_1_1dump__writer.html]

Stan: stan::io::dump\_writer Class Reference


|  |  |
| --- | --- |
|  | Stan  2.5.0  probability, sampling & optimization |


- Main Page
- Related Pages
- Namespaces
- Classes
- Files

- Class List
- Class Index
- Class Hierarchy
- Class Members

All Classes Namespaces Files Functions Variables Typedefs Enumerations Enumerator Friends Macros Pages

- stan
- io
- dump\_writer

Public Member Functions |
List of all members

stan::io::dump\_writer Class Reference

Writes data into the S-plus dump format.
More...

`#include <dump.hpp>`

|  |  |
| --- | --- |
| Public Member Functions | |
|  | dump\_writer () |
|  | Construct a dump writer writing to standard output. More... |
|  | |
|  | dump\_writer (std::ostream &out) |
|  | Construct a dump writer writing to the specified output stream. More... |
|  | |
|  | ~dump\_writer () |
|  | Destroy this writer. More... |
|  | |
| template<typename T > | |
| void | write (const std::string &name, const T &x) |
|  | Write a variable with the specified name and value. More... |
|  | |
| template<typename T > | |
| void | dump\_structure (std::string, std::vector< size\_t > dims, std::vector< T > xs) |
|  | Write a structure variable with the specified name, dimensions, and integer or double values. More... |
|  | |
| template<typename T > | |
| void | dump\_list (std::string name, std::vector< T > xs) |
|  | Write a list variable with the specified name and integer or double values. More... |
|  | |
| template<typename T > | |
| void | dump\_var (std::string name, T x) |
|  | Write a list variable with the specified name and integer or double values. More... |
|  | |

## Detailed Description

Writes data into the S-plus dump format.

A `dump_writer` writes data into the S-plus dump format, a human-readable ASCII representation of arbitrarily dimensioned arrays of integers and arrays of floating point values.

Definition at line 42 of file dump.hpp.

## Constructor & Destructor Documentation

|  |  |  |  |  |
| --- | --- | --- | --- | --- |
| stan::io::dump\_writer::dump\_writer | ( |  | ) |  |

inline

Construct a dump writer writing to standard output.

Definition at line 272 of file dump.hpp.

|  |  |  |  |  |  |
| --- | --- | --- | --- | --- | --- |
| stan::io::dump\_writer::dump\_writer | ( | std::ostream & | *out* | ) |  |

inline

Construct a dump writer writing to the specified output stream.

Parameters
:   |  |  |
    | --- | --- |
    | out | Output stream for writing. |

Definition at line 280 of file dump.hpp.

|  |  |  |  |  |
| --- | --- | --- | --- | --- |
| stan::io::dump\_writer::~dump\_writer | ( |  | ) |  |

inline

Destroy this writer.

Definition at line 285 of file dump.hpp.

## Member Function Documentation

template<typename T >

|  |  |  |  |
| --- | --- | --- | --- |
| void stan::io::dump\_writer::dump\_list | ( | std::string | *name*, |
|  |  | std::vector< T > | *xs* |
|  | ) |  |  |

inline

Write a list variable with the specified name and integer or double values.

Parameters
:   |  |  |
    | --- | --- |
    | name | Name of variable. |
    | xs | Values of variable. |

Template Parameters
:   |  |  |
    | --- | --- |
    | T | `double` or `int`. |

Definition at line 340 of file dump.hpp.

template<typename T >

|  |  |  |  |
| --- | --- | --- | --- |
| void stan::io::dump\_writer::dump\_structure | ( | std::string | , |
|  |  | std::vector< size\_t > | *dims*, |
|  |  | std::vector< T > | *xs* |
|  | ) |  |  |

inline

Write a structure variable with the specified name, dimensions, and integer or double values.

name Name of variable.

Parameters
:   |  |  |
    | --- | --- |
    | dims | Dimensions of variable. |
    | xs | Values of variable in last-index major format. |

Template Parameters
:   |  |  |
    | --- | --- |
    | T | `double` or `int`. |

Definition at line 322 of file dump.hpp.

template<typename T >

|  |  |  |  |
| --- | --- | --- | --- |
| void stan::io::dump\_writer::dump\_var | ( | std::string | *name*, |
|  |  | T | *x* |
|  | ) |  |  |

inline

Write a list variable with the specified name and integer or double values.

Parameters
:   |  |  |
    | --- | --- |
    | name | Name of variable. |
    | x | Value of variable. |

Template Parameters
:   |  |  |
    | --- | --- |
    | T | `double` or `int`. |

Definition at line 355 of file dump.hpp.

template<typename T >

|  |  |  |  |
| --- | --- | --- | --- |
| void stan::io::dump\_writer::write | ( | const std::string & | *name*, |
|  |  | const T & | *x* |
|  | ) |  |  |

inline

Write a variable with the specified name and value.

This method will work for basic types consisting of doubles and integers, Eigen types for vectors, row vectors and matrices. If this method works for a type, it will work for a standard vector of that type.

Information concerning final type organization into Eigen vectors, row vectors and matrices is lost.

All data is written in final-index dominant ordering.

Parameters
:   |  |  |
    | --- | --- |
    | name | Name of variable to write. |
    | x | Value of variable. |

Template Parameters
:   |  |  |
    | --- | --- |
    | Type | of variable being written. |

Definition at line 306 of file dump.hpp.

---

The documentation for this class was generated from the following file:

- src/stan/io/dump.hpp


---

[ Stan Home Page ]

*© 2011–2014,
Stan Development Team.*
